# Study of light-induced MscL gating by EPR spectroscopy

**DOI:** 10.1007/s00249-015-1063-4

**Published:** 2015-08-19

**Authors:** Duygu Yilmaz, Anna I. Dimitrova, Martin Walko, Armagan Kocer

**Affiliations:** Department of Biochemistry, University of Groningen, Nijenborgh 4, 9747 AG Groningen, The Netherlands; Department of Neuroscience, University Medical Center Groningen, University of Groningen, Antonius Deusinglaan 1, 9713 AV Groningen, The Netherlands

**Keywords:** Mechanosensitive channel of large conductance, Protein engineering, EPR, Synthetic biology, Interaction parameter

## Abstract

A number of techniques developed to investigate protein structure and function depend on chemically modifying and/or labeling of proteins. However, in the case of homooligomeric proteins, the presence of multiple identical subunits obstructs the introduction of residue-specific labels to only one or several subunits, selectively. Here, in order to study the initial conformational changes of a homopentameric mechanosensitive ion channel during its gating, we developed a method for labeling a defined number of subunits of the channel with two different cysteine-specific compounds simultaneously. The first one is a light-sensitive channel activator that determines the degree of openness of the ion channel upon irradiation. The second one is a spin label, containing an unpaired electron, which allows following the resulting structural changes upon channel gating by electron paramagnetic resonance spectroscopy. With this method, we could open MscL into different sub-open states. As the number of light switches per channel increased, the intersubunit spin–spin interactions became less, indicating changes in intersubunit proximities and opening of the channel. The ability of controlled activation of MscL into different open states with a noninvasive trigger and following the resulting conformational changes by spectroscopy will pave the way for detailed spectroscopic studies in the area of mechanosensation.

**Electronic supplementary material:**

The online version of this article (doi:10.1007/s00249-015-1063-4) contains supplementary material, which is available to authorized users.

## Introduction

**Fig. 1:**
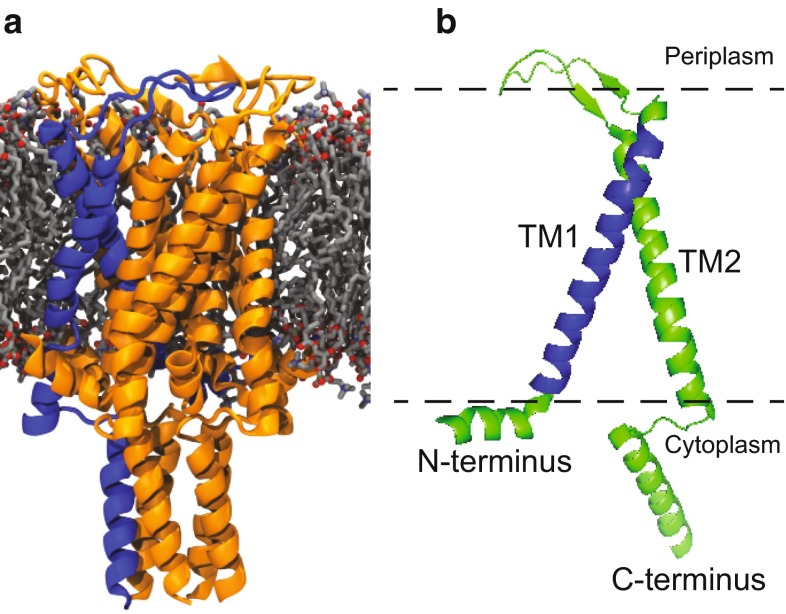
Structure of MscL. **a** Side view of MscL along the membrane plane. MscL figure is aligned to the *E. coli* sequence and based on the crystal structure of pentameric wild-type MscL from *Mycobacterium tuberculosis* (Steinbacher et al. 2007). **b** Schematic presentation of an MscL monomer. The *dotted line* marks the cellular membrane (*TM* transmembrane)

MscL is the first bacterial mechanosensitive channel to be cloned and sequenced (Martinac et al. 1987; Sukharev et al. 1994). To date, *Escherichia coli* MscL has been one of the best-characterized mechanosensitive channels. Crystal structure of MscL was obtained from *Mycobacterium tuberculosis* (Chang et al. 1998) in a closed/nearly closed state and revealed MscL as a homopentamer of approximately 15-kDa subunits, each of which contains 136 amino acid residues (Chang et al. 1998) (Fig. 1a). Each MscL subunit consists of two transmembrane domains, designated TM1 and TM2, separated by a periplasmic loop and flanked by amino-terminal and carboxy-terminal α-helices (Chang et al. 1998) (Fig. 1b). TM1 helices form a tightly packed bundle funneling to a pore constriction of ~2 Å, which expands up to 30 Å upon channel opening (Cruickshank et al. 1997; Wang et al. 2014). Despite a substantial body of data, the information on the initiation of mechanosensation is still missing.

Electron paramagnetic resonance (EPR) spectroscopy in combination with site-directed spin labeling (SDSL) has emerged as a powerful method for studying the structure and conformational dynamics of membrane proteins under conditions relevant to function (Hubbell et al. 1996, 1998, 2000; Klug and Feix 1998; Steinhoff 2002). In this technique, a spin-label side chain is introduced at a selected site of the protein via modification of the cysteine sulfhydryl group with a paramagnetic nitroxide reagent. The continuous wave (CW) EPR spectrum yields information about the nitroxide side chain mobility, solvent accessibility, the polarity of its immediate environment, and the distance between the nitroxide and another paramagnetic center in the protein. EPR-SDSL was used to study the gating mechanism of many ion channels (Perozo et al. 1998), including MscL (Perozo et al. 2002a, b). In these studies, Perozo and coworkers trapped MscL at undefined intermediate states during its transition from the closed to the fully open state by reconstituting the channel into lipids with different acyl chain lengths or by changing the bilayer properties through addition of a non-bilayer forming lipid (l-α-lysophosphatidylcholine, LPC). These studies provide invaluable information about the channel gating, but in case of MscL they suffered from a particular limitation. When protein oligomers with multiple spin labels (as MscL channel) are considered for EPR study, the proximity between spin labels of identical subunits leads to spin exchange and/or dipolar coupling. Consequently, this affects the overall line shape of the EPR spectra and compromises the analysis.

**Fig. 2:**
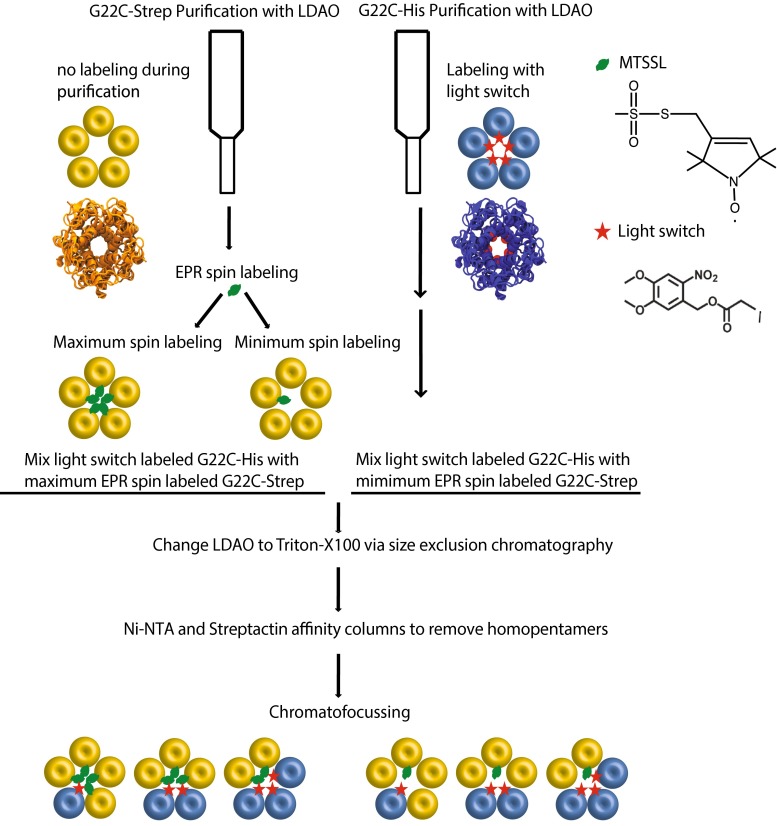
Method of generating heteropentamers of MscL with defined number of light switches and EPR spin labels

Here, we present an approach that enables activation of MscL into initial sub-open states in a well-controlled manner by using light. We labeled MscL in its hydrophobic pore region on an engineered cysteine residue at the G22 position using a cysteine-selective alkylating reagent. The reagent is composed of an iodoacetate bearing the photocleavable protecting group 6-nitroveratryl alcohol (Fig. 2, Kocer et al. 2005). This compound is sensitive to long-wavelength ultraviolet. Upon illumination at *λ* > 300 nm, the photolysis of the protective group leaves negatively charged cysteine-bound acetates at the pore of MscL, which activated the channel with hydrophobic gating mechanism (Birkner et al. 2012). During the channel opening, we monitor the resulting structural changes on MscL by employing spin-label EPR spectroscopy. We believe that controlled activation of MscL into defined sub-open states from the initiation of its pore opening would help understanding the initiation of mechanosensation process at the molecular level. Furthermore, the method also offers labeling the desired number of monomers within a pentamer. Therefore, it can also be used to avoid the dipolar spin and spin–exchange interactions.

## Methods

### Plasmid and strain

As previously described (Birkner et al. 2012), *E. coli mscL* gene was encoded in the p1BAD vector, and protein expression was performed in an *mscL*-null *E. coli* PB104 strain. The p1BAD vector provides ampicillin resistance, a 6His-tag or Strep-II tag for purification, and arabinose control over protein expression. Site-directed mutagenesis was accomplished by polymerase chain reaction, using QuikChange site-directed mutagenesis kit (Stratagene, La Jolla, CA, USA). Briefly, wild-type MscL in p1BAD was used as a template, and oligonucleotide primers were designed that incorporated the desired codon change accompanied by two 12- to 18-base pair flanking sequences on each side. All mutants were verified by DNA sequencing and DNA restriction analysis.

### Protein expression and purification

Mutant proteins were expressed as described previously (Kocer et al. 2007; Birkner et al. 2012). In short, all mutant constructs were transformed into CaCl_2_-competent *E. coli* PB104 cells and were grown in Luria–Bertani medium in the presence of 10 μg/ml chloramphenicol and 100 μg/ml ampicillin. Cells were grown in a bioreactor at pH 7.5, temperature 37 °C, and oxygen control (dissolved oxygen >70 %), using a complex medium [12 g/l bacto-tryptone (BD), 24 g/l yeast extract (BD), potassium phosphate (17 mM KH_2_PO_4_ and 72 mM K_2_HPO_4_) (pH 7), supplemented with chloramphenicol and ampicillin]. Forty percent (vol/vol) glycerol 10 ml/l medium was used as additional carbon source and 0.1 % (w/v) l-arabinose to induce the protein expression. Cells were harvested after 120 min.

Membrane vesicles were prepared as described elsewhere (Kocer et al. 2007). Briefly, cells were broken using a cell disrupter (Type TS/40; Constant Systems) at 1.7 kbar and 5 °C. After two subsequent centrifugation steps, membrane vesicles were resuspended and homogenized in ice-cold 25 mM Tris–HCl (pH 8.0) to 0.7 g (wet weight)/m, and frozen in liquid nitrogen and stored at −80 °C.

### Protein isolation

#### Isolation of 6His-tagged protein

Protein was isolated as described by Kocer et al. with minor modifications (Kocer et al. 2007). Membrane vesicles were solubilized in solubilization buffer (10 mM sodium phosphate buffer pH 8.0, 300 mM NaCl, 1 % (v/v) LDAO and 35 mM imidazole) and insoluble material was removed by ultracentrifugation. The solubilized fraction was then applied to Ni–NTA agarose resin (Qiagen), which was pre-equilibrated with solubilization buffer. After 30-min incubation, the unbound material was allowed to drain, and the column was washed with 15 column volume (CV) of wash buffer (10 mM sodium phosphate (pH 8.0), 300 mM NaCl, 0.2 % (vol/vol) LDAO, 35 mM imidazole). Then the column was further washed with 50 mM histidine buffer (wash buffer containing 50 mM histidine). Finally, the protein was eluted with 235 mM histidine buffer (wash buffer containing 235 mM histidine) and the fractions were analyzed for protein content by the Bradford assay.

#### Treatment of MscL with different denaturants

Purified MscL fractions were separately treated with 2 % (w/v) trifluoroethanol (TFE), 4 % (w/v) TFE, 4 M urea, 6 M urea, 4 M guanidinium hydrochloride, 6 M guanidinium hydrochloride, and 0.6 % (w/v) sodium dodecyl sulfate (SDS). After incubation for 1 h at room temperature, the samples were loaded in blue native gel.

### Blue native gel electrophoresis

Blue native gel electrophoresis was carried out according to the method of Schägger and von Jagow (1991). Briefly, 8–16 % gradient acrylamide gels were cast on a Bio-Rad minigel system. Gel buffer was comprised of 500 mM aminocaproic acid and 50 mM Bis–Tris, pH 7.0. Cathode buffer contained 50 mM Tricine, 15 mM Bis–Tris, pH 7.0, 0.02 % Serva blue G-250 (w/v); and anode buffer contained 50 mM Bis–Tris pH 7.0. Sample buffer was 75 mM aminocaproic acid containing 0.3 % Serva blue G-250 (w/v). Following sample loading, the gel was run at 150 V until the front line had entered into one-third of the gel, whereupon the cathode buffer was replaced by the one that did not have Serva blue G-250 (50 mM Tricine, 15 mM Bis–Tris, pH 7.0). Gel running was then continued at 200 V until complete. After being de-stained (30 % v/v methanol, 10 % v/v acetic acid), the protein was visible as sharp Coomassie blue-stained bands.

### EPR spin labeling

By using site-directed spin labeling (SDSL) procedure, the cysteine mutants were specifically labeled with (1-oxyl-2,2,5,5-tetramethylpyrrolin-3-methyl) methanethiosulfonate (MTS, Toronto Research Chemicals Inc. (TRC), Toronto, Canada). To achieve the desired labeling, the purified protein was spin-labeled with a 10:1 or 1:10 ratio of spin label:protein (mol/mol) for maximum labeling and under-labeling, respectively. Labeling was done in detergent solution at room temperature for 30 min. After incubation, the unbound spin label was removed by employing size exclusion chromatography using commercially available NAP10 columns (GE Healthcare). The labeled protein was collected in three fractions and analyzed for the presence of the free label by EPR spectroscopy. The fraction with only labeled protein was used in further experiments.

#### Isolation of StrepII-tagged protein and labeling with light switch

Protein was isolated as described by Birkner et al. with minor modifications (Birkner et al. 2012). Membrane vesicles were solubilized by solubilization buffer (10 mM sodium phosphate buffer pH:8.0, 300 mM NaCl, 1 % (v/v) LDAO). The solubilized fraction was then applied to streptactin resin (IBA), which was equilibrated with solubilization buffer. After 30-min incubation, the unbound material was allowed to drain, and the column was washed with 15 CV of wash buffer (10 mM sodium phosphate (pH 8.0), 300 mM NaCl, 0.2 % (vol/vol) LDAO). Then the column matrix was incubated with the light switch (Fig. 2, Kocer et al. 2005) dissolved in DMSO (2 mg/ml). After 45 min at room temperature, the column was washed further with wash buffer in order to remove excess light switch label. Finally, the protein was eluted with 10 mM biotin and the fractions were analyzed for protein content by the Bradford assay.

### Generation and separation of heteropentamers

6His-tagged and StrepII-tagged proteins were mixed in 1:1 (v/v) ratio. After incubation at room temperature for 45 min, the mixture was applied to size exclusion column (equilibrated with 10 mM NaPi pH 8.0, 300 mM NaCl, 0.2 % (w/v) Triton X-100) in order to change the detergent to Triton X-100. The fractions obtained from the size-exclusion column were separated into individual heteropentameric proteins in a three-step procedure as explained before (Birkner et al. 2012). Our strategy was to eliminate the homopentameric MscL channels in two consecutive affinity chromatography steps and separate the resulting heteropentameric mixture into individual proteins by chromatofocusing. Briefly, the protein mixture containing the homo- and hetero-pentameric MscL channels was applied first to Ni–NTA affinity column. While all proteins having at least one his-tag binds to this column, the StrepII tagged homopentamers were eliminated in the flow-through fraction. The Ni–NTA-bound proteins were then eluted and applied to the second affinity column Streptactin. While all heteropentamers with at least one Strep-tag binds to the matrix, the 6-His-tagged homopentamers were eliminated in the flow-through fraction. The eluent containing all heteropentamers of MscL was separated into its individual heteropentamers by chromatofocussing as described before (Birkner et al. 2012). Briefly, a Mono P 5/200 GL chromatofocussing column (GE Healthcare) was equilibrated with 20 ml of start buffer (25 mM Tris–acetic acid (pH 8.3) at 10 °C, 10 mM NaCl, 0.2 % (vol/vol) Triton X-100), and a pre-gradient was formed by washing the column with 6 ml of elution buffer pH 5.0 (7 ml of Polybuffer 74, 3 ml Polybuffer 96, 10 mM NaCl, 0.2 % (vol/vol) Triton X-100). The heteropentamer mixture was desalted prior to application to the chromatofocussing column by using a NAP-10 column. During the elution, 250-μl fractions were collected. The pH of the fractions was determined at 6 °C. The protein content of the peak fractions was determined by using a 2-D Quant Kit (GE Healthcare) according to the manufacturer’s protocol.

### Protein reconstitution into liposomes

Proteins were reconstituted into synthetic liposomes according to Kocer et al. (2007). Briefly, lipid suspension (asolectin) was homogenized by extrusion 11 times through a 400-nm filter. Liposomes were saturated by the addition of Triton X-100. Protein and lipids were mixed at 1:35 weight ratio and incubated for 30 min at 50 °C. Subsequently, the mixture was supplemented with 6 mg (wet weight) Biobeads (SM-2 Absorbents; Bio-Rad) per microliter of detergent (10 % Triton X-100) used in the sample and lipid preparation. For detergent removal, the sample was incubated overnight (ca. 16 h) at 4 °C under mild agitation. The next morning, liposomes were pelleted down by ultracentrifugation for EPR measurements.

#### EPR spectroscopy

Continuous wave (CW) EPR measurements were performed using a commercially available MiniScope benchtop X-band EPR spectrometer (MS400 Magnettech GmbH, Berlin, Germany) with a rectangular TE102 resonator. Due to heat production in the resonator during operation, the cavity was fluxed with gaseous nitrogen, keeping the temperature stable. The microwave power was set to 10 mW, and the B-field modulation amplitude to 0.20 mT. After determining the overmodulation range of Miniscope 400, i.e., *B*_m_ ≤ 50 % _Δ_*B*_pp_ (where, *B*_m_ is the modulation amplitude, and _Δ_*B*_pp_ is the peak-to-peak first-derivative line width), to get the highest signal intensity we set the modulation amplitude (*B*_m_) to 43 % of the narrowest line width. EPR glass capillaries (0.9 mm inner diameter) were filled with a sample volume of 50 µl, and the final protein concentration was in the range of 220–285 µM. The microwave frequency was 9.41 GHz, and the modulation frequency was 100 kHz. Each spectrum corresponds to 25 or 36 accumulations (5 min total measurement time) for maximally and minimally spin-labeled protein, respectively.

### Spin labeling efficiency

The spin labeling efficiency (SLE) was calculated by comparison of the area of the EPR absorption spectra of the labeled protein and a reference spin probe (MTSSL) of known concentration by using Eq. (1):

1 $$C_{\text{samp}} = \frac{{N_{\text{samp}} \cdot C_{\text{ref}} }}{{N_{\text{ref}} }}$$

where *C*_samp_ is the concentration of the spin label in the protein sample, *N*_samp_ and *N*_ref_ are the evaluated integral areas of the absorption spectra for the sample and the reference, respectively, and *C*_ref_ is the reference concentration. The obtained *C*_samp_ was used to calculate the SLE by using Eq. (2), where *C*_prot_ is the protein concentration (determined by standard Bradford assay).

2 $${\text{SLE = }}\frac{{C_{\text{samp}} }}{{C_{\text{prot}} }}$$

### EPR data analysis

To determine the interaction parameter, the area under the derivative EPR signal was used. First, the signal-to-noise ratio for the spectrum of each labeled protein was improved by subtracting a background spectrum, which was generated by measuring the reconstituted protein without EPR spin-label (25 and 36 scans were accumulated for the maximally and minimally labeled protein, respectively). Next, the area under the derivative EPR signal was determined by double integration. After each integration, a baseline correction was performed using Magnettech software, and a good baseline on the low- and high-field sides of the spectrum was obtained. The y-axis of the second integral was proportional to the number of spins in the measured probe. The original intensity of the spectrum was divided by it, and each spectrum was plotted with the new intensity amplitude.

## Results and discussion

In order to activate MscL into defined sub-open states and follow resulting conformational changes, we generated wild-type-G22C MscL heteropentamers bearing two different labels per pentamer, i.e., a light switch and an EPR spin label. Previously, by cloning both wild-type and G22C MscL independently of each other in the same *E. coli* cells, and a three-step purification procedure, we could generate MscL heteropentamers WT_n_-G22C_(5−n)_, where *n* is the number of subunits (Birkner et al. 2012). We could control MscL opening by changing the hydrophobicity of the pore lining residue G22, one subunit at a time, in WT_n_-G22C_(5−n)_ heteropentameric channels using a cysteine-specific charged compound 2-(trimethylammonium)ethyl methanethiosulfonate, Bromide (MTSET). Covalent attachment of MTSET into cysteine 22 position in each G22C-containing subunit of the heteropentamer increased the hydrophilicity of the pore and opened the channel in the absence of membrane tension. This way, individual heteropentamers could be opened only up to certain open state depending on the number of G22C subunits in a pentamer. However, this method does not serve our present purpose of labeling the same heteropentamer with a defined number of the light switch and the EPR spin, which are both cysteine-specific labels.

Therefore, our new approach was (1) dissociating MscL homopentamers into monomers; (2) labeling the monomers with light switch and/or EPR spin label; (3) mixing light-switch-labeled MscL and EPR spin-labeled MscL monomers in different ratios; (4) associating them back into pentamers; and (5) isolating pentamers with defined ratio of light-switch-labeled and EPR spin-labeled MscL monomers by chromatofocussing (Fig. 2).

**Fig. 3:**
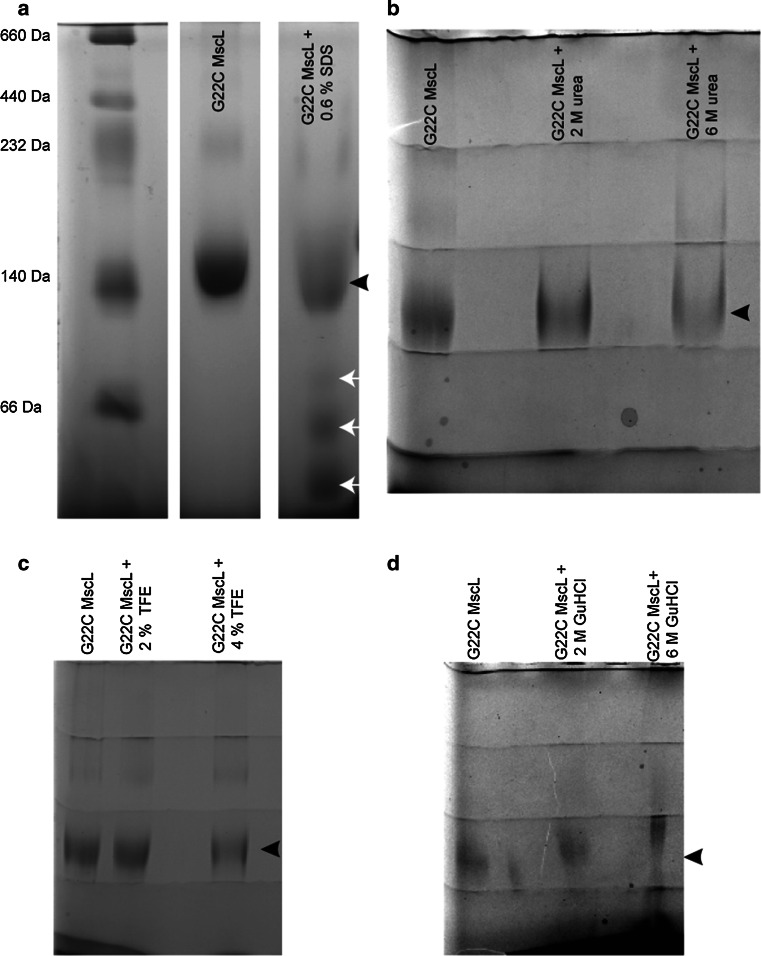
MscL treated with different denaturants ran on blue native gel. A portion of pure G22C MscL was treated with **a** SDS (final concentration 0.6 % w/v), **b** urea (final concentration 2 and 6 M), **c** trifluoroethanol (TFE) (final concentration 2 and 4 % w/v), and **d** guanidinium hydrochloride (final concentration 2 and 6 M). The *black and white arrows* show the pentameric and smaller oligomeric forms of MscL, respectively

In order to dissociate MscL pentamers into monomers, we first screened various denaturants including urea, guanidinium hydrochloride, trifluoroethanol (TFE) (Fig. 3a–d), and various detergents such as Triton X-100, C12E9, foscholine 10, nonyl-glucoside, decyl-glucoside, cymal 3, CHAPS, CHAPSO, and SDS. None of the indicated compounds could dissociate the pentamers of MscL into monomers (Fig. 3b–d, black arrow), except for SDS (Fig. 3a, white arrows). However, the removal of SDS from the channel was problematic in the further steps.

Therefore, we applied a different strategy to obtain the monomers of MscL. Previously, it has been reported that the oligomeric state of MscL differs depending on the detergent used during purification (Dorwart et al. 2010). While *n*-octylpentaoxyethylene (C_8_E_5_) and Triton X-100 allow MscL from *S. aureus* to properly fold and assemble into a pentameric structure, *n*-dodecyl-*N*,*N*-dimethylamine-*N*-oxide (LDAO) assembles MscL into tetramers. When we used LDAO during purification of *E. coli* MscL, we could detect only monomers and dimers of detergent-solubilized MscL with native mass spectrometry (Konijnenberg et al. 2014).

Next, we expressed MscL in *E. coli* and purified it from the membranes by using LDAO. After purification, we re-assembled MscL into pentamers by exchanging LDAO with the detergent Triton X-100 via size-exclusion chromatography. In order to check the re-association of MscL monomers into pentamers, we first applied the protein to chromatofocussing and determined its p*I*. We detected a single peak in chromatofocussing at a p*I* value of 6.23 (Figure S1a), which corresponds to the pentameric protein (Birkner et al. 2012). Then, we reconstituted the channel purified in LDAO and the channel after its detergent is exchanged to Triton X-100 into liposomes individually and performed the calcein dequenching assay as described by Kocer et al. (2007). When MscL purified in LDAO was reconstituted into liposomes, the channel activity, as measured by percent release was 40 %. However, when the detergent LDAO was exchanged to Triton X-100 (same protein:lipid ratio), the activity was 85 % (Figure S1b). Therefore, we anticipated that after the detergent exchange, the channels re-organize into a functional pentameric state.

The heteropentameric assemblies of MscL with a defined number of a light switch and EPR spin label per pentamer were generated as illustrated in Fig. 2. G22C MscL channels were tagged at the C-terminus with either a 6His- or a StrepII-tag at the gene level. After expression of the channels in bacteria, the homopentameric channels were purified and the StrepII-tagged MscL channels were labeled with the EPR spin label (both maximally and minimally) and the His-tagged MscL channels were labeled with the light switch. Next, light switch-labeled-StrepII-tagged MscL channels were mixed with EPR spin-labeled- His-tagged MscL channels and the detergent was changed to Triton X-100 by size exclusion chromatography. By this way, all possible combinations of homo- and hetero-pentameric assemblies of MscL channels were obtained. Homopentameric MscL channels (all subunits labeled with light switch and all subunits labeled with EPR spin label) were removed by two subsequent affinity column chromatographies. The heteropentameric channel assemblies were then separated by chromatofocusing based on their p*I* values (Figure S2a–b). The pick fractions were used in further experiments.

To study the conformational changes of individual heteropentamers, we performed EPR spectroscopy. Since 356-nm monochromatic light was used to activate the MscL opening, we first checked the effect of light irradiation on the spin label. Even though it has been shown that UV illumination at this wavelength activates the light switch while it does not affect the MscL integrity and functionality (Kocer et al. 2005), its effect on the spin label is not known. Therefore, we labeled G22C MscL with the spin label, reconstituted it into asolectin liposomes, and irradiated for 300 s at 30-s intervals. Comparison of EPR spectra before and after irradiation reveals no spectroscopic changes between non-irradiated protein and protein irradiated for 300 s, showing that irradiation for 300 s does not affect spin label attached to the protein or the protein itself (Figure S3).

After defining the optimum experimental conditions for EPR spectroscopy, we studied the channel gating using heteropentameric MscL with 1, 2, and 3 light switches per pentamer. We have described (Birkner et al. 2012) that the presence of one charge per pentamer leads to gating of MscL to initial sub-open states in its journey from the closed to fully open state and increasing number of charges per pentamer increases the probability of gating to higher sub-open states. Using variable amount of light switches together with the spin labeling thus allowed us to observe changes in the channel in various sub-open states. In EPR, we followed the channel opening by measuring the intersubunit spin–spin interaction parameter, Ω, as described before (Perozo et al. 1998, 1999). Ω gives a rough estimation of intersubunit proximities by analyzing the spectral broadening caused by spin–spin interactions.

**Fig. 4:**
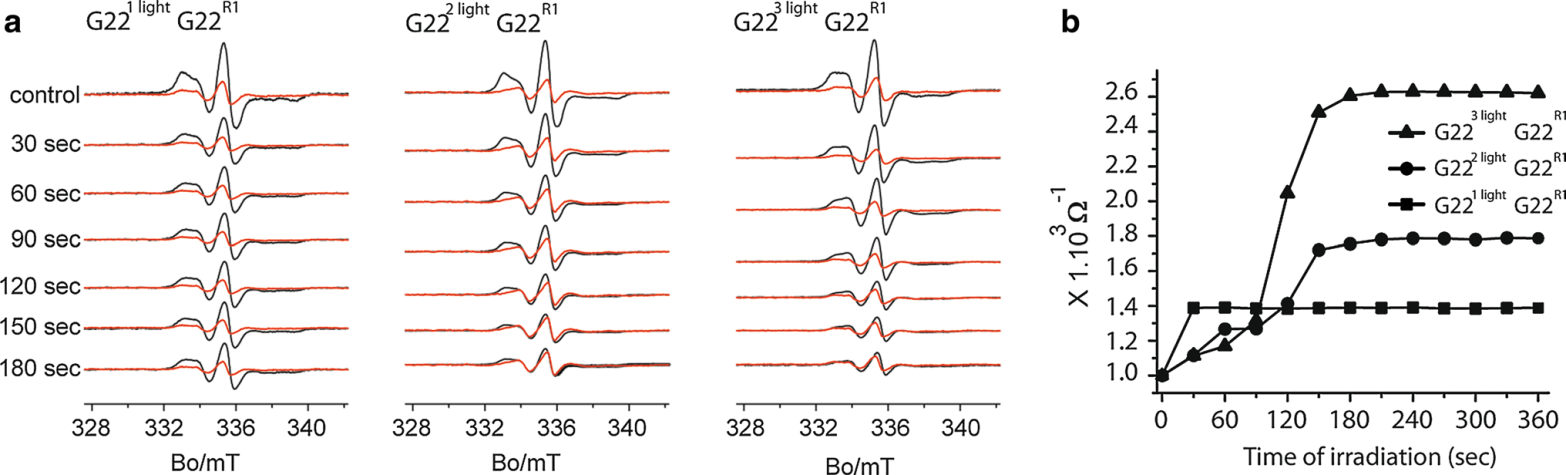
**a** Room temperature CW-EPR of G22C MscL with 1 (*left panel*), 2 (*middle panel*), and 3 (*right panel*) light switches per pentamer irradiated by light. Traces *in black* were obtained from maximally labeled channels (10:1 molar ratio, spin label/subunit). Traces *in red* were obtained from under-labeled channels (1:10 molar ratio, spin label/subunit). **b** Inverse value of the interaction parameter (Ω^−1^) as a function of the irradiation time. Data for G22C MscL with 1, 2, or 3 light switches per pentamer

In each condition, we used both maximally and minimally EPR spin-labeled sample. The spin labeling efficiency (SLE) for minimally labeled protein was 9 %. The SLE for maximally labeled MscL channels with one, two, and three light switches were 68, 50, and 33 %, respectively. As shown in Fig. 4a, black traces correspond to maximally labeled channels, while red traces represent the minimally labeled channels. In the maximally spin-labeled mutant, all available cysteines were modified with MTSSL spin label. Spin-labeled monomers can either be next to or opposite to each other. In both conditions, as a result of close proximity of the spin labels, the spin–spin interactions lead to an EPR spectral broadening. In the minimally spin-labeled channels, the vast majority of the channels are expected to have a single nitroxide group and hence, the spectra should be free of spin–spin interactions. Next, we quantified this broadening by the difference of the inverse values of the interaction parameter: Ω^−1^, which is extracted from the extent of the spectral broadening of a maximally EPR spin-labeled sample relative to one with no spin–spin interactions (minimally EPR spin-labeled sample). Ω^−1^ is calculated as the ratio of amplitudes of the central resonance line (mI = 0) between the maximum labeled and the under-labeled mutant, both normalized to the total number of spins. As we illuminate the sample, light irradiation induces channel opening (Kocer et al. 2005), which results in an increase in the distance between the paramagnetic centers and hence increase in Ω^−1^ value (Fig. 4b).

As shown in Fig. 4b, when there is one light switch present per pentamer, the first 30-s irradiation induces a small change in Ω^−1^ value, while further irradiation does not cause any changes (Fig. 4b, squares). In the presence of two light switches per pentamer, the increase in Ω^−1^ value to almost twice its original value after 180 s of irradiation demonstrates larger spin–spin distance and is associated with increased channel opening as a result of additional light-induced charge inside the protein (Fig. 4b, circles). When three light switches are present per pentamer, a further increase in the spin–spin distance is observed as indicated by the increase in Ω^−1^ value, indicating an even bigger channel opening (Fig. 4b, triangles). After 180-s irradiation time, no further EPR spectral changes were observed, resulting in a plateau in the function [Ω^−1^ = *f* (time)] (Fig. 4b).

Having observed that the biggest change in the interaction parameter occurs when there are three light switches present per pentameric G22C MscL, we next investigated other residues along TM1 by using three light switches per pentamer. We chose residue 24 and 28 for their importance in the gating of the channel (Perozo et al. 2002a, b). In EPR experiments, we maintained the position of the light switch at the 22nd position, and changed the position of the EPR spin label to 24th and 28th positions along the TM1.

**Fig. 5:**
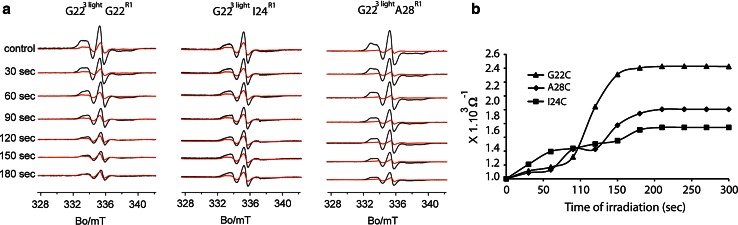
**a** Room-temperature CW-EPR of MscL labeled with EPR spin label at position number 22nd (*right panel*), 24th (*middle panel*), and 28th (*right panel*) showing different degrees of spectral broadening due to spin–spin coupling. Traces *in black* were obtained from fully labeled channels (10:1 molar ratio, spin label/subunit). Traces *in red* were obtained from under-labeled channels (1:10 molar ratio, spin label/subunit). **b** Inverse values of the interaction parameter (Ω^−1^) as a function of the time of irradiation

Figure 5a shows the different degrees of spectral broadening due to channel opening for these residues. We quantified the line broadening by using the inverse values of the interaction parameter, Ω^−1^ again. Figure 5b shows the inverse values of the interaction parameter as a function of time of irradiation for the selected residues I24C and A28C. We observed a similar Ω^−1^ value for all the residues after 90 s of irradiation, although the trend of the increase was different for each residue. At the end of 180 s of irradiation, the biggest increase in Ω^−1^ was observed for G22C, followed by A28C and I24C, respectively. Based on our results, we conclude that when there are three light switches present per pentamer, among the three residues we selected, G22C position is subjected to the biggest change during channel gating.

In conclusion, we developed a method to generate heteropentameric MscLs from wild-type and G22C MscL subunits by dissociating and re-associating MscL subunits. We isolated heteropentamers with a defined number of subunits labeled with different cysteine-specific compounds: the light switches and EPR spin labels. Finally, we tested the feasibility of this approach for investigating the early conformational changes taking place during channel opening by examining three positions: G22C, I24C, and A28C, and showed that it is possible to follow the gating of MscL in real time by activating the channel gradually and non-invasively by light and monitoring the resulting structural changes by EPR. In the very first and through EPR study on the closed state of MscL (Perozo et al. 2001), the G22, I24, and A28 positions showed larger broadening than what we observed in our work. This might be explained by closer spins in these positions in the absence of the bulky light switch. In the case of partially open states of MscL, Perozo et al. (2002a, b) could trap MscL at unknown intermediate open states by successfully reconstituting MscL into synthetic phosphatidylcholine lipids containing monosaturated acyl chains of 16 carbons and at an open state by using lysophosphatidylcholine. By comparing the maximally and minimally labeled MscL, they quantified the broadening in the segment between G22 and G26. As compared to this data, activating MscL with up to three light switches per channel, generated a milder increase in the spectral mobility that that obtained 16C lipid and also LPC, suggesting that light switch might activate the channel to initial subopen states but not to the fully open state as LPC does.

Finally, the method provides a reliable approach for overcoming the limitation imposed by the fivefold symmetry of the MscL in spectroscopic studies. Even though we employed EPR in this particular work, the controlled activation of the channel with a non-invasive trigger serves a valuable tool to study early conformational changes of MscL by various spectroscopic techniques.

## **Electronic supplementary material**


**Figure S1 a** Chromatofocussing profile of G22C-Strep MscL after detergent exchange from LDAO to Triton X-100 **b** Calcein dequenching assay of reconstituted MscL, following purification in LDAO and after the detergent exchange to Triton X-100 **(PNG 76 kb)**

**Figure S2** Heteropentamers of MscL separated by chromatofocussing. The heteropentamers are either **(a)** minimally labeled with EPR spin label, or **(b)** maximally labeled with EPR spin label **(PNG 142 kb)**

**Figure S3** Effect of irradiation on R1 spin label **(PNG 32 kb)**
