# Supplementary figures and images for: Study of light-induced MscL gating by EPR spectroscopy
Source: Eur Biophys J. 2015 Aug 19;44(7):557–65. doi: 10.1007/s00249-015-1063-4 (PMC4562997; doi:10.1007/s00249-015-1063-4)

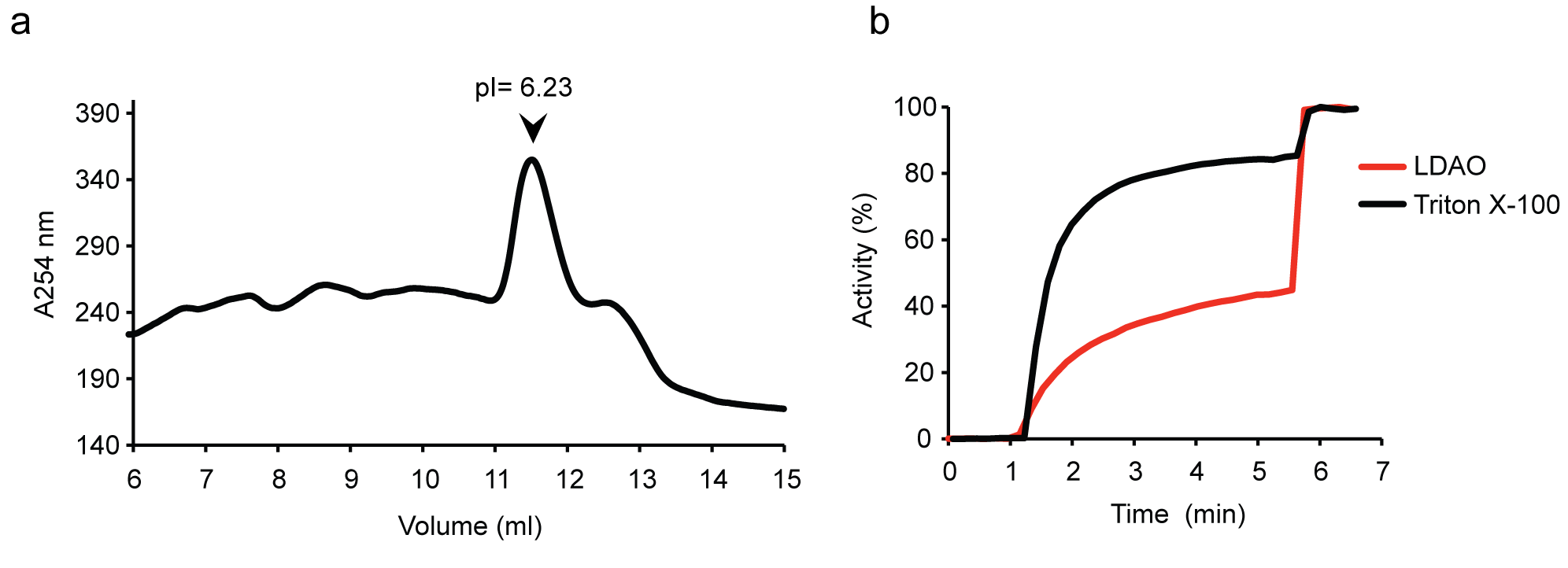

Supplement: Supplementary file 1 — Figure S1 a Chromatofocussing profile of G22C-Strep MscL after detergent exchange from LDAO to Triton X-100 b Calcein dequenching assay of reconstituted MscL, following purification in LDAO and after the detergent exchange to Triton X-100 (PNG 76 kb) [file 249_2015_1063_MOESM1_ESM.png]

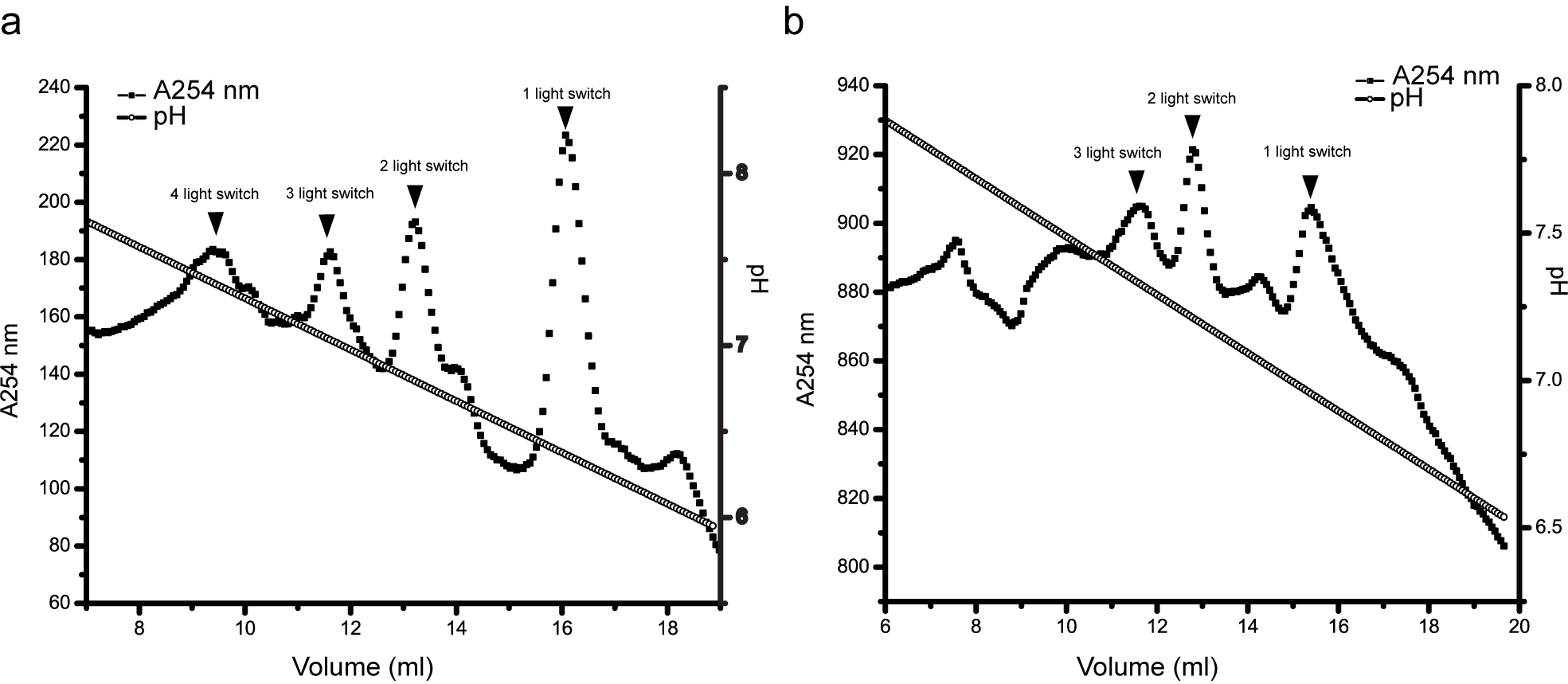

Supplement: Supplementary file 2 — Figure S2 Heteropentamers of MscL separated by chromatofocussing. The heteropentamers are either (a) minimally labeled with EPR spin label, or (b) maximally labeled with EPR spin label (PNG 142 kb) [file 249_2015_1063_MOESM2_ESM.png]

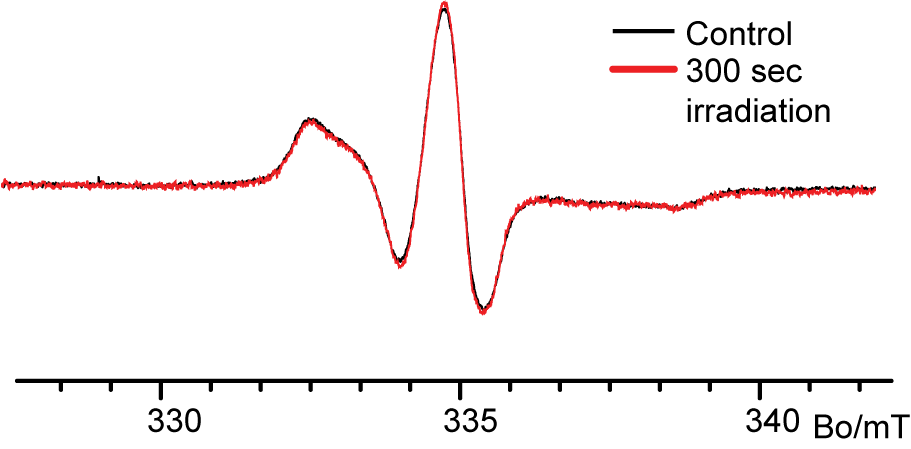

Supplement: Supplementary file 3 — Figure S3 Effect of irradiation on R1 spin label (PNG 32 kb) [file 249_2015_1063_MOESM3_ESM.png]
